# Sleep disturbances and predictors of nondeployability among active-duty Army soldiers: an odds ratio analysis of medical healthcare data from fiscal year 2018

**DOI:** 10.1186/s40779-020-00239-7

**Published:** 2020-03-10

**Authors:** Jaime K. Devine, Jacob Collen, Jake J. Choynowski, Vincent Capaldi

**Affiliations:** 1 Institutes for Behavior Resources, Operational Fatigue and Performance, 2104 Maryland Ave, Baltimore, MD 21218 USA; 2 Pulmonary, Critical Care and Sleep Medicine Walter Reed National Military Medical Center, Bethesda, MD 20889 USA; 3 Behavioral Biology Branch, Center for Military Psychiatry and Neuroscience, Walter Reed Army Institute of Research, Silver Spring, MD 20910 USA

**Keywords:** Medical readiness, Behavioral sleep medicine, Deployability, Healthcare records, Military, Big data, Data mining

## Abstract

**Background:**

The impact of sleep disorders on active-duty soldiers’ medical readiness is not currently quantified. Patient data generated at military treatment facilities can be accessed to create research reports and thus can be used to estimate the prevalence of sleep disturbances and the role of sleep on overall health in service members. The current study aimed to quantify sleep-related health issues and their impact on health and nondeployability through the analysis of U.S. military healthcare records from fiscal year 2018 (FY2018).

**Methods:**

Medical diagnosis information and deployability profiles (e-Profiles) were queried for all active-duty U.S. Army patients with a concurrent sleep disorder diagnosis receiving medical care within FY2018. Nondeployability was predicted from medical reasons for having an e-Profile (categorized as sleep, behavioral health, musculoskeletal, cardiometabolic, injury, or accident) using binomial logistic regression. Sleep e-Profiles were investigated as a moderator between other e-Profile categories and nondeployability.

**Results:**

Out of 582,031 soldiers, 48.4% (*n =* 281,738) had a sleep-related diagnosis in their healthcare records, 9.7% (*n =* 56,247) of soldiers had e-Profiles, and 1.9% (*n =* 10,885) had a sleep e-Profile. Soldiers with sleep e-Profiles were more likely to have had a motor vehicle accident (pOR (prevalence odds ratio) =4.7, 95% CI 2.63–8.39, *P ≤* 0.001) or work/duty-related injury (pOR = 1.6, 95% CI 1.32–1.94, *P ≤* 0.001). The likelihood of nondeployability was greater in soldiers with a sleep e-Profile and a musculoskeletal e-Profile (pOR = 4.25, 95% CI 3.75–4.81, *P ≤* 0.001) or work/duty-related injury (pOR = 2.62, 95% CI 1.63–4.21, *P ≤* 0.001).

**Conclusion:**

Nearly half of soldiers had a sleep disorder or sleep-related medical diagnosis in 2018, but their sleep problems are largely not profiled as limitations to medical readiness. Musculoskeletal issues and physical injury predict nondeployability, and nondeployability is more likely to occur in soldiers who have sleep e-Profiles in addition to these issues. Addressing sleep problems may prevent accidents and injuries that could render a soldier nondeployable.

## Background

Readiness is the number one priority in the U.S. Army [1]*.* The Army’s ability to restore and regenerate equipment, expertise and personnel are key to its ongoing success [2]*.* Soldiers who are unable to deploy when the Army needs them directly affect this readiness. Reducing the rate of nondeployability among soldiers is a complex challenge that Army organizations and senior staff need to address to maximize readiness [3]*.*

Decreasing the number of nondeployable soldiers across the Army depends on determining and mitigating the underlying causes for nondeployable conditions. Soldiers can be categorized as nondeployable due to administrative, legal or medical conditions. Notably, medically nondeployable soldiers constitute the largest category, accounting for approximately 80% of nondeployable soldiers [3, 4]. Medical readiness, therefore, is an important target for reducing nondeployability in the Army.

Understanding which medical conditions most greatly contribute to nondeployability is essential in developing strategies for preventative care and risk mitigation. The Army maintains a centralized authoritative database of medical readiness information of Army personnel known as the Medical Occupational Data System (MODS). Within the MODS are two Web-based modules that track and record medical readiness information. The Medical Protection System (MEDPROS) is the primary tool to record, track, and report soldiers’ medical conditions, and the electronic profiling system (e-Profile) tracks whether any medical conditions may render soldiers medically unable to deploy on a temporary or permanent basis [5, 6]. Soldiers can acquire a “profile” in six different categories: physical functional capacity (P), upper extremities (U), lower extremities (L), hearing and ears (H), eyes and vision (E), and psychiatric (S). Together, the six categories are often referred to as “PULHES”. Soldiers with e-Profiles have a score between 1 and 4 for each PULHES category. A score of 1 indicates that the soldier is medically sound in that category, while a score of 2 indicates a mild impairment (i.e., a soldier with an E (eyes and vision) score of 2 may require glasses). A permanent e-Profile score of 3 or higher in any PULHES category indicates that the soldier is medically undeployable. For example, an E score of 4 may indicate blindness. Soldiers can have multiple concurrent e-Profiles for multiple medical conditions.

Sleep is a factor related to health concerns across the medical spectrum. Sleep disorders in and of themselves are debilitating [7, 8], but sleep is also related to physical and mental health [9–12]. For example, individuals with insomnia or obstructive sleep apnea (OSA) are at greater risk for obesity, type 2 diabetes, atherosclerosis, coronary heart disease, heart failure, hypertension, stroke, and trauma-related nightmares [9, 13]. Moreover, fatigue is a contributing factor to the occurrence of traffic and workplace accidents [14–17]. It is therefore possible that sleep problems could contribute to medical conditions that result in permanent e-Profiles. However, while sleep complaints and sleep disorders are common among soldiers [13, 18–21], the impact of sleep-related health issues on deployability within active-duty service members is not currently quantified.

Understanding the scope of sleep problems and their impact on deployability will help command leadership and medical professionals determine where to concentrate efforts to treat and prevent debilitating health issues among service members and maintain the medical readiness of the U.S. Army. The current study aimed to quantify the prevalence of sleep-related health issues through the analysis of U.S. military healthcare records from fiscal year 2018 (FY2018) to assess the relationship between sleep disorders and health and nondeployability in active-duty soldiers.

## Methods

Patients data generated through the MODS, MEDPROS and e-Profile are stored in the Composite Healthcare System (CHCS). The Standard Inpatient Data Record (SIDR) is extracted from the CHCS database twice per month and transmitted securely to the Patient Administration Systems and Biostatistics Activity (PASBA). The Comprehensive Ambulatory/Professional Encounter Record (CAPER) is extracted from the CHCS database daily and transmitted securely to PASBA. The data in the PASBA can be accessed by submitting a data extraction request and data sharing agreement to the Defense Health Agency (DHA) and the Office of the Surgeon General (OTSG) for research and for decision support activities to enable the U.S. Army Medical Command to operate effectively.

**Fig. 1:**
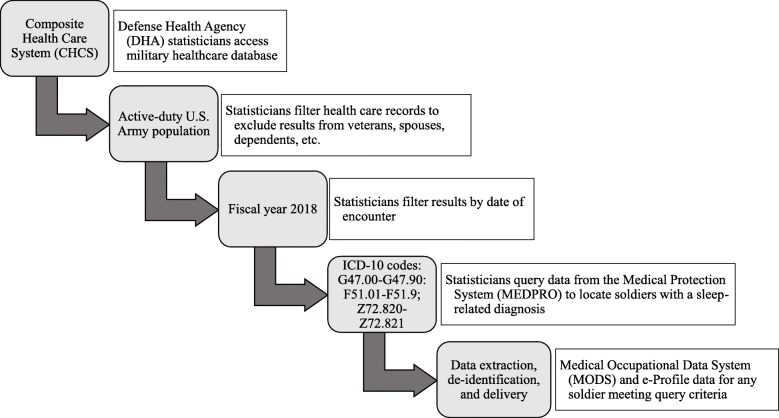
Data extraction flow chart. Visualization of the data extraction process by which active-duty Army soldier medical healthcare records were queried by Defense Health Agency statisticians and delivered to researchers for the current analyses

Data for the current study were requested from, de-identified and compiled by a PASBA DHA health statistician prior to delivery/analysis, as depicted in Fig. 1. Medical diagnosis information and e-Profile status data were queried for all active-duty U.S. Army patients with a concurrent sleep disorder diagnosis receiving medical care within fiscal year 2018. Sleep disorders were queried using International Statistical Classification of Diseases and Related Health Problems, version 10 (ICD-10) medical diagnosis codes in the following ranges: G47.00-G47.9: Sleep disorder; F51.01-F51.9: Sleep disorders not due to a substance or known physiological condition; and Z72.820-Z72.821: Problems related to sleep. A fictional linking identifier was created to replace Department of Defense medical identifiers for all patient information. Data were compiled using Excel 2013 (Microsoft Corp., Redmond, WA, USA) and Access 2013(Microsoft Corp., Redmond, WA, USA).

SPSS statistical software (version 25, SPSS, Inc., Chicago, IL, USA) was used to conduct statistical analysis of de-identified e-Profile data. An alpha level of 0.05 was used for all statistical tests. Binary variables were calculated from the e-Profile classification system called “reason for visit” such that sleep e-Profiles were defined as “reason for visit = sleep or focus area: sleep”, behavioral health e-Profiles were defined as “reason for visit = behavioral health” (with the exception of sleep disorders, which were categorized under sleep e-Profiles) and cardiometabolic health e-Profiles were defined as “reason for visit = cardiology, endocrine/general, or gastroenterology”. Soldiers with any e-Profile (temporary or permanent, all scores) due to that condition were categorized as having the condition (coded as 1), while soldiers with only e-Profiles for any other medical condition were categorized as not having the condition (coded as 0).

Binary variables for all causes of nondeployability, nondeployability due to a sleep disorder, nondeployability due to behavioral health, and nondeployability due to cardiometabolic health were calculated from PULHES scores such that all temporary e-Profiles and permanent e-Profiles with scores ≤2 in all categories were considered deployable and a permanent e-Profile PULHES score ≥ 3 was considered nondeployable. Additionally, medical records due to motor vehicle accidents (defined as injury mechanism: traffic accident or motor vehicle accident) and work/duty-related injuries (defined as injury mechanism: battle injury, duty-related injury, or work−/task-related injury) were used to create binary variables for e-Profiles due to accident or work/duty-related injury.

Chi-squared tests for independence examined the relationship between e-Profiles for sleep, behavioral health, cardiometabolic health, work-related injuries and accidents and deployability. *P* values were considered significant when p was equal to or less than 0.05. Binomial logistic regressions predicted the likelihood of nondeployability for soldiers who had an e-Profile due to sleep, behavioral health, cardiometabolic health, work-related injury or an accident compared to those who did not, as well as the likelihood of having an e-Profile for behavioral health, cardiometabolic health, work/duty-related injury or motor vehicle accident for soldiers with or without an e-Profile for sleep using block entry [22]. Further, interaction effects between sleep e-Profiles and other variables of interest (behavioral health, cardiometabolic health, work/duty-related injury or motor vehicle accidents) were calculated to examine moderation of the relationship between predictors of nondeployability and sleep e-Profiles.

## Results

### Soldier and e-profile descriptives

There were on average 582,031 active-duty Army soldiers in FY2018. Of this active-duty population, 48.4% (*n =* 281,738) soldiers fit the search criteria of having sought medical treatment for a sleep-related condition (as defined by ICD-10 codes) in FY2018 and 9.7% (*n =* 56,247) had a concurrent e-Profile tracking a temporary or permanent medical conditions that may have rendered them medically not ready to deploy. Soldiers with e-Profiles ranged from 17 to 66 years old (mean ± SD: 36.5 ± 9.4; median: 37) and the average body mass index (BMI) was 29.0 ± 4.1 kg/m^2^. The majority of soldiers with e-Profiles were male (71.3%, *n =* 40,080).

About 99 % (99.7%, *n =* 56,082) of soldiers had seven e-Profiles or less. The remaining 0.3% (*n =* 165) of soldiers had between 8 and 20 e-Profiles. Musculoskeletal conditions (such as injury to the spine, bones or joints) were the most commonly listed condition for e-Profiles, accounting for 71.8% (*n =* 40,508) of all e-Profiles. Sleep disorders were the listed condition for 19.4% (*n =* 10,885) of all e-Profiles from soldiers with sleep-related diagnoses in FY2018. Specific diagnoses associated with sleep e-Profiles are summarized in Table 1.

**Table 1:** Sleep disorder diagnoses in soldiers with e-Profiles

Listed condition	Case	Percentage of soldiers with sleep e-Profile (%)	Percentage of soldiers with Sleep-related diagnosis (%)	Percentage of active-duty population (%)
Sleep e-Profile (All)	10,885	100.0	19.4	1.9
Obstructive sleep apnea e-Profiles	10,442	96.0	3.7	1.8
Narcolepsy and circadian disorder e-Profile	190	1.7	0.07	0.03
Insomnia e-Profile	107	1.0	0.04	0.02
Narcolepsy and OSA e-Profiles	30	0.3	0.01	0.005

Breakdown of sleep e-Profiles by listed condition for active-duty soldier populations from FY2018. OSA was the listed condition for the majority of soldiers with sleep e-Profiles and represented 1.9% of all active-duty soldiers

About 16 % of soldiers with e-Profiles (16.2%, *n =* 9119) were classified as nondeployable. The leading reason for nondeployability was musculoskeletal conditions, accounting for 47.9% (*n =* 4366) of nondeployability. Behavioral health was the listed reason for nondeployability in 27.2% (*n =* 2481) of nondeployable soldiers. Cardiometabolic health was the listed reason for nondeployability in 8.1% (*n =* 736) of the nondeployable soldiers. Sleep disorders were the listed reason for nondeployability in 1.8% (*n =* 165) of nondeployable soldiers. The listed reasons for nondeployability in the remaining 15.0% (*n =* 1371) of soldiers were related to a wide range of health conditions, such as urological, pulmonary, and degenerative conditions.

### Prevalence odds ratios for comorbid conditions in relation to sleep e-profiles

The comorbidity of sleep e-Profiles and musculoskeletal e-Profiles, cardiometabolic e-Profiles, and behavioral health e-Profiles, as well as the prevalence of motor vehicle accidents, work/duty-related injuries and nondeployability, are summarized in Table 2. Comorbidities of sleep profiles were statistically significant for all e-Profile types (*P <* 0.05) except for behavioral health (*P =* 0.34). Soldiers with sleep e-Profiles were more likely to also have had a motor vehicle accident or work/duty-related injury compared to soldiers without a sleep e-Profile.

**Table 2:** Prevalence odds ratios of having a sleep e-Profile and other e-Profile category, accident, injury, and nondeployability

Item	Sleep e-Profile [*n* (%)]		pOR	95%CI	*P*-Value
	Yes	No			
Nondeployable					
Yes No	2027(3.6)	7092(12.6)	0.81	0.77–0.85	*P ≤* 0.001
	8858(15.8)	38,270(68.0)			
Musculoskeletal e-Profile					
Yes	6740(12.0)	34,470(61.2)	0.51	0.49–0.54	*P ≤* 0.001
No	4145(7.4)	10,892(19.4)			
Cardiometabolic e-Profile					
Yes	46(0.1)	1322(2.5)	0.67	0.60–0.75	*P ≤* 0.001
No	10,418(19.0)	44,040(78.4)			
Behavioral health e-Profile					
Yes	1040(1.8)	4470(8.0)	1.04	0.96–1.11	*P =* 0.34
No	9845(17.5)	40,892(72.7)			
Motor vehicle accident					
Yes	12(0.1)	234(0.4)	4.7	2.63–8.39	*P ≤* 0.001
No	10,873(19.3)	45,128(80.2)			
Work/duty-related injury					
Yes	120(0.2)	794(1.4)	1.6	1.32–1.94	*P ≤* 0.001
No	10,765(19.1)	44,568(79.3)			

Prevalence odds ratio analysis of the likelihood of comorbidity between sleep e-Profiles and other e-Profile types. Soldiers with sleep e-Profiles were more likely to have a musculoskeletal e-Profile, motor vehicle accident, or work/duty-related injury than soldiers without a sleep e-Profile

Prevalence odds ratios for nondeployability in relation to having a musculoskeletal, cardiometabolic or behavioral health e-Profile, motor vehicle accident or work/duty-related injury are summarized in Table 3. Nondeployability was significantly related to all e-Profile types (all *P ≤* 0.001; data not shown). Nondeployable soldiers were significantly more likely to have musculoskeletal e-Profiles or have had a work/duty-related injury. In addition, there was also a trend (*P* = 0.06) for nondeployable soldiers to have had a motor vehicle accident.

**Table 3:** Prevalence odds ratios of nondeployability and profile categories, motor vehicle accidents and work/duty-related injuries

Item	Nondeployable [*n*(%)]		pOR	95%CI	*P*-value
	Yes	No			
Musculoskeletal profile					
Yes	6174(11.0)	34,234(60.9)	1.27	1.21–1.33	*P ≤* 0.001
No	2945(5.2)	12,894(22.9)			
Cardiometabolic profile					
Yes	851(1.5)	938(1.7)	0.2	0.18–0.22	*P ≤* 0.001
No	8268(14.7)	46,190(82.1)			
Behavioral health profile					
Yes	2894(5.1)	2616(4.6)	0.13	0.12–0.13	*P ≤* 0.001
No	6225(11.1)	44,512(79.1)			
Motor vehicle accident					
Yes	29(0.1)	217(0.4)	1.45	0.98–2.14	*P =* 0.06
No	9090(16.1)	46,911(83.4)			
Work/duty-related injury					
Yes	109(0.2)	805(1.4)	1.44	1.17–1.76	*P ≤* 0.001
No	9010(16.0)	46,323(82.4)			

Prevalence odds ratio analysis of the likelihood of being nondeployable and having a musculoskeletal, cardiometabolic or behavioral health e-Profile, motor vehicle accident or work/duty-related injury. Nondeployable Soldiers were significantly more likely to have musculoskeletal e-Profiles or have had a work/duty-related injury

The moderation effects of having a sleep e-Profile on the odds ratios for nondeployability were examined for musculoskeletal e-Profiles, cardiometabolic e-Profiles, behavioral health e-Profiles, motor vehicle accidents and work/duty-related injuries. Table 4 summarizes the interaction between sleep e-Profiles and the three variables found to predict greater odds of nondeployability (musculoskeletal e-Profiles, motor vehicle accidents and work/duty-related injuries). Soldiers with a musculoskeletal e-Profile and a sleep e-Profile were significantly more likely to be nondeployable, and soldiers with a work/duty-related injury and sleep e-Profile were nearly three times as likely to be nondeployable. The odds ratio of nondeployability given that a soldier had an incidence of a motor vehicle accident in addition to a sleep e-Profile was not statistically significant.

**Table 4:** Prevalence odds ratios of nondeployability, predictors of nondeployability and sleep e-Profiles

Item		Nondeployable [*n*(%)]		pOR	95%CI	*P*-value
		Yes	No			
Musculoskeletal e-Profile and Sleep e-Profile						
	Yes	1565(2.8)	5116(9.1)	4.25	3.75–4.81	*P ≤* 0.001
	No	7554(13.4)	42,012(74.7)			
Motor Vehicle Accident and Sleep e-Profile						
	Yes	3(0.0)	9(0.0)	2.16	0.55–8.52	*P =* 0.27
	No	9116(16.2)	47,119(83.8)			
Work/Duty-Related Injury and Sleep e-Profile						
	Yes	31(0.1)	89(0.2)	2.62	1.63–4.21	*P ≤* 0.001
	No	9088(16.1)	47,039(83.6)			

Prevalence odds ratio analysis comparing the likelihood of having a sleep e-Profile in combination with either a musculoskeletal e-Profile, motor vehicle accident or work/duty-related injuries. Soldiers with a musculoskeletal e-Profile and a sleep e-Profile or a work/duty-related injury and a sleep e-Profile were more likely to be nondeployable than soldiers with only one or neither of those conditions

## Discussion

Nearly half of active-duty U.S. Army soldiers had a diagnosis for a sleep-related issue during fiscal year 2018. However, only 3.8% of these soldiers additionally had an e-Profile for their sleep condition. This may indicate that sleep disorders are not generally considered by military clinicians to impact medical readiness to a degree sufficient to warrant the generation of an e-Profile. Even when profiled, soldiers with a sleep, cardiometabolic, or behavioral health e-Profile were less likely to be nondeployable. These statistics indicate that sleep, cardiometabolic and behavioral health disorders do not greatly influence the medical readiness and deployability of U.S. Army soldiers.

In contrast, musculoskeletal issues accounted for 47.9% of the nondeployability of soldiers. Soldiers who had a motor vehicle accident or work/duty-related injury were also more likely to be nondeployable (Table 3). Interestingly, soldiers with sleep e-Profiles were over one and a half times more likely to also experience accidents or injuries as soldiers with no sleep e-Profile.

Subsequently, we examined the moderation of predictors of nondeployability by comorbid sleep e-Profiles. Soldiers with a musculoskeletal e-Profile or work/duty-related injury in addition to a sleep e-Profile were between two to four times more likely to be nondeployable as soldiers with either one or none of these issues (Table 4). The odds ratio for having a both motor vehicle accident and sleep e-Profile and being nondeployable was not significant, most likely due to the small sample size of soldiers with an e-Profile for motor vehicle accidents (*n =* 246). While it is impossible to determine causal relationships between predictors of nondeployability and sleep from these data, the picture begins to emerge that sleep disorders are connected to medical readiness in indirect and undocumented ways, such as through correlation with injuries.

Interestingly, having a sleep e-Profile was not related to an increased likelihood of having a behavioral health e-Profile. This finding contradicts the known relationship between mental health and sleep from the literature [10, 19, 23]. Additionally, soldiers with sleep e-Profiles were less likely to have a cardiometabolic profile (Table 2). The current data pertain to medical diagnoses or medical readiness information as determined by a military clinician. Many cardiometabolic and behavioral health disorders are comorbid with sleep disorders or have sleep disturbances as a symptom [9, 19, 24–27]. This overlap in symptomology may not translate to having an e-Profile when clinicians are asked to categorize a soldier’s limitations to medical readiness using the PULHES scoring system. Additionally, considering that soldiers with e-Profiles for sleep, cardiometabolic or behavioral health issues were less likely to be nondeployable, it is possible that these conditions are not generally considered serious enough to limit a soldier’s ability to serve. For example, OSA, which accounted for the vast majority of sleep disorder diagnoses in the current study, can be treated with oral devices, surgery or positive airway pressure devices [28] and thus would not necessarily limit a soldiers’ functionality.

Additionally, an underappreciation for the severity of sleep problems may prevent soldiers from discussing fatigue-related issues with their healthcare providers. While the Department of Defense recognizes sleep as an important component of health and the performance triad [29], Army culture has not traditionally shared this respect [30]. Undervaluing the need for sleep likely contributes to the prevalence of sleep disturbances in active-duty soldiers through poor sleep hygiene but may also mask the severity of sleep problems in soldiers who downplay the importance of rest or who think that complaining about sleep may be interpreted as a sign of weakness.

It should be noted that the e-Profile data extracted for these analyses were only from active-duty soldiers with a sleep-related diagnosis in fiscal year 2018. Therefore, even though the sleep disorders accounted for e-Profiles in only 19.4% of soldiers with e-Profiles, the entire population (*n =* 281,738) from these analyses suffered from some sleep-related health issue in 2018. It is therefore unclear whether the findings from the current analyses can be generalized to the entire active-duty Army population or whether only motor vehicle accidents, work/duty-related injuries and musculoskeletal issues are predictors of nondeployability in soldiers with sleep problems. Future analyses will compare soldiers with sleep disorders to soldiers with no sleep complaints.

The current analyses are limited not only by the population but also by the source of the data. Medical healthcare data are a largely untapped resource for examining the complex manifestation of health issues, but the MODS, MEDPRO, e-Profile, SIDR or CAPER merely track medical encounters; they are not designed for hypothesis testing. As data mining techniques and the field of data science advance, there are bound to be improvements in the modeling of medical healthcare data for research needs. The interpretation of findings is limited by the disproportionate numbers of e-Profiles due to different causes, which may be indicative of actual prevalence but is less than ideal for hypothesis testing, such as the high prevalence of musculoskeletal e-Profiles (*n =* 40,508) versus the relative rarity of e-Profiles due to motor vehicle accident (*n =* 246). Moreover, the current analyses should be considered correlative rather than causative.

Despite limitations to the analyses and interpretation, the data suggest that sleep disorders are a prodigious issue in the U.S. Army. A soldier is 4.7 times more likely to have a motor vehicle accident if he or she also has a profiled sleep disorder as a soldier with no sleep e-Profile. Importantly, a soldier was more likely to be nondeployable if he or she jointly had a musculoskeletal e-Profile and a sleep e-Profile than if he or she had only one of the two e-Profiles. These numbers highlight the impact of sleep on seemingly unrelated medical issues. A recent study by Shattuck et al. likewise showed an association between musculoskeletal complaints and shorter nighttime sleep as well as an increased report of fatigue in crewmembers on a U.S. Navy aircraft carrier [31], indicating that the comorbidity of sleep and musculoskeletal issues may be an epidemic across the Armed Services.

## Conclusions

In addition to half of active-duty soldiers being diagnosed with sleep-related issues, sleep seems to be negatively impacting the medical readiness of service members. Musculoskeletal issues and physical injury predict nondeployability, and the occurrence of these problems is related to having a sleep disorder. Sleep disturbances constitute an underlying risk to medical readiness. Decreasing risk to active-duty military should go beyond the obvious causes and address underlying issues such as sleep disturbances to improve medical readiness and maximize the health of all U.S. service members.

## Data Availability

The datasets generated and/or analyzed during the current study are not publicly available due to the potential sensitive nature of military healthcare data.

## Abbreviations

BMI: Body mass index
CAPER: Comprehensive ambulatory/professional encounter record
CHCS: Composite healthcare system
DHA: Defense health agency
E-Profile: Electronic profiling system
FY2018: Fiscal year 2018
ICD-10: International statistical classification of diseases and related health problems, version 10
MEDPROS: Medical protection system
MODS: Medical occupational data system
OSA: Obstructive sleep apnea
OTSG: Office of the surgeon general
PASBA: Patient administration systems and biostatistics activity
POR: Prevalence odds ratio
PULHES: Physical functional capacity (P), upper extremities (U), lower extremities (L), hearing and ears (H), eyes and vision (E), and psychiatric (S)
SIDR: Standard Inpatient Data Record.
